# Mouth breathing is associated with a higher prevalence of anterior dental caries in preschool children

**DOI:** 10.1590/1807-3107bor-2024.vol38.0057

**Published:** 2024-12-09

**Authors:** Maria Eliza da Consolação Soares, Joana Ramos-Jorge, Laura Jordana Santos Lima, Luana Viviam Moreira, Izabella Barbosa Fernandes, Maria Letícia Ramos-Jorge, Rodrigo Galo

**Affiliations:** a Universidade Federal dos Vales do Jequitinhonha e Mucuri - UFVJM, School of Biological and Health Sciences, Department of Dentistry, Diamantina, MG, Brazil.; b Universidade Federal de Minas Gerais - UFMG, School of Dentistry, Department of Child and Adolescent Oral Health, Belo Horizonte, MG, Brazil.; c Universidade São Paulo – USP, , Department of Dental Materials and Prosthodontics, Ribeirão Preto, SP, Brazil.

**Keywords:** Dental Caries, Mouth Breathing, Child, Preschool

## Abstract

This cross-sectional study aimed to investigate the association between anterior dental caries and the predominant type of breathing in preschoolers. The research involved a sample of 257 children aged between 3 and 5 years, who were enrolled in public daycare centers and preschools in the city of Diamantina, MG, Brazil. A questionnaire was applied to parents/guardians to collect sociodemographic data, habits, oral health, and a dietary diary used to calculate the Sucrose Consumption Index. The predominant type of breathing, whether nasal or through the mouth, was determined through direct observation. Dental caries was assessed using the International Caries Detection and Assessment System (ICDAS). Descriptive analyses and the Poisson regression were conducted to analyze the data. The results revealed a higher prevalence of anterior dental caries among children who predominantly breathed through the mouth (PR = 1.57; 95%CI: 1.01–2.46; p = 0.047), who exhibited a higher frequency of sucrose consumption (PR = 4.02; 95%CI: 2.03–7.95; p < 0.001), and had mothers with lower educational levels (PR = 1.53; 95%CI: 1.01–2.32; p = 0.043). Pacifier use was associated with a lower prevalence of anterior dental caries (PR = 0.57; 95%CI: 0.34–0.96; p = 0.035). In conclusion, this investigation demonstrated an association between carious lesions in the anterior teeth and predominantly mouth breathing in preschoolers.

## Introduction

Dental caries is a complex disease involving the activity of bacteria within the oral biofilm and is markedly influenced by sugar intake. It has multiple causal factors, and results in the loss, and subsequent recovery, of minerals present in dental hard tissues.^1^ When this decay affects the primary teeth of a child under 6 years old, it is termed Early Childhood Caries (ECC).^2^ The prevalence of ECC is notably high in this age group, reaching approximately 46.2% globally.^3^ Socioeconomic factors, bottle feeding, dietary patterns, and oral hygiene habits have been identified as associated elements contributing to its heightened prevalence in preschoolers.^4-9^

Another factor explored as a potential contributor to childhood caries is predominantly mouth breathing, which could lead to a reduction in salivary flow and subsequent decrease in oral cavity humidity.^10^ However, findings on this matter are inconsistent.^11-14^ This investigation holds significance, given that mouth breathing ranks among the most prevalent deleterious oral habits in children, with a prevalence ranging from 11% to 57% globally.^10,15,16^ The occurrence of mouth breathing can be linked to obstructions in various locations within the upper airway.^10^

Simulated night-time mouth breathing in adults has been shown to cause a reduction in intraoral pH during sleep compared to predominantly nasal breathing. Therefore, it has been suggested that this phenomenon may pose a risk factor for erosion and caries.^17^ Additionally, the connection between mouth breathing and gingivitis in anterior teeth, even in the absence of bacterial biofilm, is attributed to a decrease in salivary flow.^18^ The combination of reduced salivary flow and low pH levels may enhance the demineralization caused by the metabolites produced by microorganisms in the presence of fermentable carbohydrates.

The characteristics of anterior deciduous teeth, namely small size, wide pulp chamber, and relatively thin enamel, all contribute to a smaller surface area for bonding during restorative procedures.^19^ Consequently, restorative treatment becomes challenging and can be further complicated by difficulties in handling a child's behavior. Moreover, the rise in pollution observed in recent years has led to an increased prevalence of respiratory diseases and mouth breathers, particularly in children.^20^ In this scenario, investigating the potential association between caries and breathing becomes crucial to guide the undertaking of preventive measures. In addition, this association underscores the significance of collaboration between pediatric dentists and other healthcare professionals, including otorhinolaryngologists and speech therapists, for early diagnosis and treatment of both conditions. The aim of the present study was thus to assess the association between the predominant type of breathing and the presence of caries lesions in the anterior teeth of preschool children. The study hypothesis was that children who predominantly breathe through the mouth would exhibit a higher prevalence of caries in anterior teeth.

## Methods

This study was conducted following the guidelines for Strengthening the Reporting of Observational Studies in Epidemiology (STROBE).^21^

### Ethical considerations

This study was approved by the Ethics Committee for Research on Human Beings, Federal University of Vales do Jequitinhonha e Mucuri (Diamantina, MG, Brazil; CAAE: 43295315.4.0000.5108). Informed consent forms were signed by all parents or guardians, and the research adhered to the ethical principles outlined in the Declaration of Helsinki.

### Sample and study design

This cross-sectional study took place from February to June 2015 and involved children aged 3 to 5 years who were enrolled in public daycare centers and preschools in Diamantina, MG, Brazil. A preliminary pilot study with 30 children was conducted to inform the calculation of the sample size for the main study. The main study excluded children who participated in the pilot study, and its sample size was calculated considering a prevalence of 42% of mouth breathers with caries and 25% of nasal breathers with caries (as observed in the pilot study). The statistical power was set at 80%, with a standard error of 5%. The minimum sample size required was 240 children. To account for potential losses, 10% was added to the calculation, resulting in a total of 260 children for the main study.

Data collection was conducted at all nine public educational institutions catering to preschoolers. A simple draw was carried out to select students from each school. The exclusion criteria were neurological disorders such as cerebral palsy or Down syndrome. Children exhibiting flu or cold symptoms on the examination day were assessed at a later time, once the signs and symptoms had subsided.

### Non-clinical data collection

Prior to the collection of clinical data, questionnaires were sent to the children's parents. Sociodemographic information was acquired, encompassing details such as the child's sex, age, maternal educational level (categorized as ≥ 8 years of schooling or < 8 years of schooling), and monthly family income (≥ 2 minimum wages or < 2 minimum wages). Additional data covered oral habits (use of pacifier and bottle), dental variables, such as whether the child had previously visited a dentist, and daily brushing frequency (brushing ≥ once a day or not brushing every day). Furthermore, a dietary diary was completed by the parents, documenting the foods consumed by the child over a span of three days.

The dietary diary underwent evaluation to compute the Sucrose Consumption Index (SCI). This index gauged food retention and the timing of ingestion. Concerning retention, foods scored 1 when they were liquid and/or non-retentive, or 2 when they were solid and/or retentive. Regarding the timing of ingestion, a score of 1 was assigned when consumed during main meals (breakfast, lunch, or dinner), and 2 when taken during snacks between the main meals. The score for retention at each meal was multiplied by the score for the time of ingestion. If more than one type of sucrose-containing food was consumed at a given meal, the one with the highest retention was considered. The scores for all the meals of the three-day period were summed, and the total value was divided by 3 to ascertain the average daily value. A score equal to or less than seven indicated a non-cariogenic diet, whereas a score greater than seven was deemed cariogenic.^22^ The aforementioned variables were regarded as potential confounding factors.

### Breathing examination

The breathing examination was conducted by an examiner who underwent theoretical training and a calibration process before undertaking the main study, which included evaluating 30 children aged between 3 and 5 years. Kappa values for both inter-examiner (compared with a reference standard) and intra-examiner agreement after a 15-day interval were determined to be above 0.90.

The predominant type of breathing was determined using the direct observation method, wherein the child was seated in front of the examiner for 5 minutes. During this period, the examiner observed which type of breathing predominated. Children who spent most of the time with their mouths open and exhibited at least one of the characteristics listed below were classified as mouth breathers. The characteristics commonly associated with mouth breathing observed in all children were tongue with raised dorsum and lowered tip; tongue on the floor of the mouth or interposed anteriorly between the arches; thick and everted lower lip; hyperfunction of the mentalis muscle; sagging lips, tongue, and cheeks; atypical swallowing; facial asymmetries; noisy breathing; increase in face height; maxillary atresia; malocclusion; narrow or ogival palate.^23^

### Oral clinical examination

The oral examination was conducted by an examiner who received theoretical training and underwent a calibration process before the main study, involving the assessment of 30 children aged 3 to 5 years. Inter-examiner (in comparison to a gold standard) and intra-examiner kappa values measured after a 15-day interval exceeded 0.80 for the evaluated oral conditions. Prior to the clinical examination, the children participated in supervised toothbrushing to facilitate an accurate diagnosis of the lesions.

The examination took place in a room provided by the school administration, utilizing equipment that included a headlamp (PETZL, Tikka XP, Crolles, France), a mouth mirror (PRISMA, São Paulo, Brazil), a WHO probe (Golgran Ind. and Com Ltda, São Paulo, Brazil), and gauze for drying the teeth.

The International Caries Detection and Assessment System (ICDAS II) served as the method for assessing the presence of carious lesions. This system employs codes ranging from 0 to 6, each corresponding to a specific lesion stage.^24^ Due to the unavailability of a dental air syringe for surface drying, code 1 was not taken into account. The maxillary and mandibular incisors and canines were evaluated, and the teeth with codes 2 to 6 were classified as decayed.

The presence of malocclusion was defined following the criteria proposed by Foster and Hamilton.^25^ This assessment considered an anterior open bite when there was no vertical overlapping of the incisors; posterior crossbite when the maxillary molars were in a more lingual position than the mandibular molars; anterior crossbite when the mandibular incisors were positioned ahead of the maxillary incisors; and accentuated overjet when the distance from the mandibular incisors to the incisal edge of the maxillary incisors was greater than or equal to 3 mm. All assessments were performed with teeth in occlusion. Malocclusion was evaluated as a possible confounding factor, since it is plausible that certain occlusion characteristics may modify the interaction between the biofilm and the tooth surface, facilitating retention and hindering removal, thus potentially favoring the occurrence of caries.^26^

### Data analysis

The data were analyzed using the Statistical Package for the Social Sciences program (SPSS for Windows, version 22.0, SPSS Inc., Chicago, USA). Initially, the frequencies of the studied variables were determined. The primary dependent variable was the presence of dental caries, and the primary independent variable was the predominant breathing pattern. Other independent variables were collected as potential confounding factors. The chi-square test was employed to examine the association between the independent variables and the presence of anterior dental caries. Univariate and multivariate Poisson regression analyses utilizing the stepwise method were conducted to establish the association between the experience of dental caries and the independent variables. Variables with a p-value < 0.20 in the univariate analysis were included in the multivariate analysis. Following adjustment, the final model estimated the prevalence ratio (PR) and 95% confidence interval (CI) for the selected variables.

## Results

Out of the initially selected 260 children, 257 (98.8%) participated throughout the study. The primary reason for these dropouts was the lack of cooperation from the children during the assessments (Figure).

**Figure:**
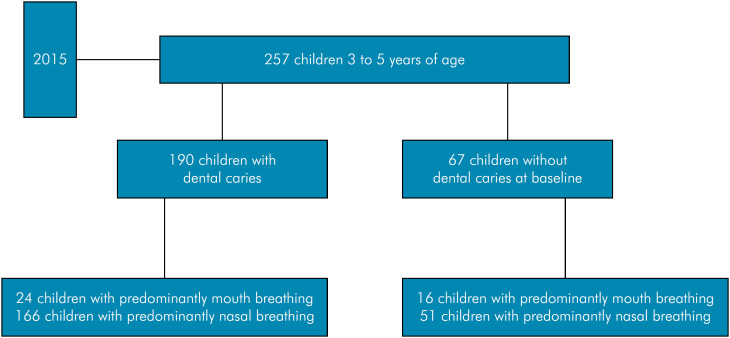
Flowchart of the study.

The mean age of the children was 4.1 years (± 0.7), and 54% (n = 139) of them were female. The prevalence of anterior dental caries was 26.1% (n = 67), and that of predominantly mouth breathing was 15.6% (n = 40).

Table 1 shows the sample's characteristics in terms of the presence or absence of anterior dental caries (dependent variable). Statistically significant differences were observed between the groups concerning monthly family income (p = 0.008), maternal educational level (p = 0.017), Sucrose Consumption Index (p < 0.001), predominant type of breathing (p = 0.029), and the habit of using a pacifier (p = 0.028).

**Table 1:** Frequency of independent variables in children with or without anterior dental caries

Variables		Without caries	With caries	p-value*
		n (%)	n (%)	
		190 (73.9)	67 (26.1)	
Breathing				
	Nasal	166 (87.4)	51 (76.1)	0.029
	Mouth	24 (12.6)	16 (23.9)	
Sex				
	Female	103 (54.2)	36 (53.7)	0.946
	Male	87 (45.8)	31 (46.3)	
Monthly family income				
	≥ 2 minimum wages	92 (48.4)	20 (29.9)	0.008
	< 2 minimum wages	98 (51.6)	47 (70.1)	
Mother's educational level				
	≥ 8 years of schooling	145 (76.3)	41 (61.2)	0.017
	< 8 years of schooling	45 (23.7)	26 (38.8)	
Ever been to the dentist				
	Yes	88 (46.3)	34 (50.7)	0.532
	No	102 (53.7)	33 (49.3)	
Daily brushing frequency				
	≥ Once a day	175 (92.1)	58 (86.6)	0.180
	Does not brush everyday	15 (7.9)	9 (13.4)	
Pacifier				
	No	126 (66.3)	54 (80.6)	0.028
	Yes	64 (33.7)	13 (19.4)	
Bottle				
	No	144 (75.8)	54 (80.6)	0.421
	Yes	46 (24.2)	13 (19.4)	
Sucrose Consumption Index				
	≤ 7	88 (46.3)	8 (11.9)	< 0.001
	7	102 (53.7)	59 (88.1)	
Some type of malocclusion				
	No	82 (43.2)	28 (41.8)	0.846
	Yes	108 (56.8)	39 (58.2)	

* Chi-square test

The univariate Poisson regression analysis revealed that the associated variables were predominant type of breathing (p = 0.020), monthly family income (p = 0.011), maternal educational level (p = 0.015), habit of using a pacifier (p=0.038), and Sucrose Consumption Index (p < 0.001; Table 2).

**Table 2:** Univariate and multivariate Poisson regression analyses using the stepwise method of factors associated with anterior dental caries.

Variable		Not adjusted PR		Adjusted PR	
		(95% CI)	p-value*	(95% CI)	p-value*
Sex					
	Female	1			
	Male	1.01 (0.67–1.53)	0.946		
Monthly family income					
	≥ 2 minimum wages	1			
	< 2 minimum wages	1.81 (1.14–2.88)	0.011		
Mother's educational level					
	> 8 years of schooling	1		1	
	≤ 8 years of schooling	1.66 (1.10–2.49)	0.015	1.53 (1.01–2.32)	0.043
Ever been to the dentist					
	Yes	1			
	No	0.87 (0.58–1.32)	0.532		
Daily brushing frequency					
	≥ Once a day	1			
	Does not brush everyday	1.50 (0.85–2.44)	0.153		
Pacifier					
	No	1		1	
	Yes	0.56 (0.32–0.96)	0.038	0.57 (0.34–0.96)	0.035
Bottle					
	No	1			
	Yes	0.80 (0.47–1.37)	0.431		
Sucrose Consumption Index					
	≤ 7	1		1	
	>7	4.39 (2.19–8.80)	<0.001	4.02 (2.03–7.95)	< 0.001
Breathing					
	Nasal	1		1	
	Mouth	1.70 (1.08–2.66)	0.020	1.57 (1.01–2.46)	0.047
Some type of malocclusion					
	No	1			
	Yes	1.04 (0.68–1.58)	0.846		

* Univariate regression; PR: Prevalence ratio; CI: Confidence interval; p-value: Statistical significance value.

After adjustment, it was observed that the prevalence of anterior dental caries was higher among children who predominantly breathed through the mouth (PR = 1.57; 95%CI: 1.01-2.46; p = 0.047). Children with a daily SCI greater than seven (PR = 4.02; CI95%: 2.03-7.95; p < 0.001) and those with mothers having 8 years of schooling or less (PR: 1.53; CI95%: 1.01-2.32; p = 0.043) also showed a higher prevalence of caries in anterior teeth. Pacifier use was associated with a lower prevalence of anterior dental caries (PR: 0.57; 95% CI: 0.34-0.96; p = 0.035; Table 2).

## Discussion

The main finding of this study was the association between anterior dental caries and the predominant type of breathing in preschool children. Children with predominant mouth breathing had a higher prevalence of anterior dental caries compared to nasal breathers. Some studies have failed to find any association between caries and mouth breathing in children and adolescents,^12,14^ whereas one study showed that mouth breathing was a risk factor for dental caries in children, corroborating the results of the present study.^11^ The reduced salivary flow observed in mouth breathers could explain this association, since saliva evaporates when air flows through the mouth, causing a decrease in moisture in the oral cavity.^10^ Mouth breathing can cause changes to the defense mediated by saliva, such as a reduction in the associated self-cleaning effect, leading to bacterial plaque accumulation and interference with intraoral pH control.^10^ Choi et al.^17^ simulated mouth breathing in healthy participants and found a reduction in nocturnal intraoral pH, which may be associated with the occurrence of caries and dental erosion.

Another factor associated with anterior dental caries was the SCI. This is in line with current evidence, in that a high frequency of sucrose ingestion has been found to be a risk factor for the onset and development of caries.^27,28^ This can be explained by the multifactorial nature of caries, a biofilm-dependent disease modulated by the metabolization of fermentable carbohydrates in the diet, especially sucrose.^29^ This metabolization generates organic acids (e.g., lactic acid), causing a low pH, and its progression results in the loss of minerals from dental tissues.^9^

The univariate analysis associated lower monthly family income and lower maternal educational level with dental caries. This aligns with recent studies.^7,30^ However, only educational level remained in the model after adjustment. Therefore, it is suggested that low schooling may reflect a lower level of instruction about hygiene care. Previous investigations have shown that a lower educational level can have a negative impact on the understanding of recommendations and advice given by health professionals.^31,32^

Deleterious oral habits are very common in early childhood.^15^ In the present study, there was no association between bottle feeding and anterior dental caries. However, it was observed that the habit of sucking a pacifier was associated with a lower prevalence of anterior caries. This result seems to contradict the study conducted by Ciribè et al.,^33^ who found that children who used a sweetened pacifier were significantly associated with the presence of caries. In other studies, no such association was found.^34,35^ The World Health Organization (WHO) contraindicates the use of pacifiers due to their potential interference with breastfeeding practices.^36^ Based on these assumptions and on the results observed in the present study, it is suggested that a pacifier can meet the need for suction while also reducing the use of a bottle, especially at night. However, this association needs further investigation.

Malocclusion may be associated with mouth breathing^37^ and dental caries.^38^ However, the association between malocclusion and dental caries was not observed in the present investigation. Arora et al.^39^ concluded that malocclusion affects oral hygiene status; however, normal occlusion or malocclusion did not play any role in the prevalence of dental caries. The assessment of oral hygiene was only evaluated through a questionnaire on the frequency of brushing, which does not represent the effectiveness of hygiene. Thus, it may have limited the effect of hygiene on the prevalence of anterior caries in the studied sample.

The limitations of the present investigation must be addressed. The type of breathing was not diagnosed by a medical specialist, which only allowed suggesting mouth breathing based on subjective observations. The cross-sectional design of this study does not allow the causality of associations to be determined. Therefore, future studies with more robust designs may explain and clarify certain factors involved in the occurrence of dental caries, such as the role of mouth breathing. Code 1 of the ICDAS was not considered due to the unavailability of a dental air syringe for surface drying, which may have led to the underreporting of non-cavitated caries lesions. Another limitation to be considered is that the sample consisted only of children enrolled in public schools, which limits the extrapolation of results to different populations.

This study provides initial data on the association between mouth breathing and anterior dental caries, and its initial hypothesis was confirmed. This highlights the importance of closely monitoring mouth-breathing children for the possibility of developing tooth decay. Dental caries in the anterior region can significantly affect oral health–related quality of life in preschoolers.^40^ Therefore, comprehensive prevention strategies and oral health promotion initiatives are necessary. However, to obtain a more solid understanding of this relationship, comparative studies with representative samples and a longitudinal design are warranted, allowing for a more in-depth analysis over time.

## Conclusion

Preschoolers exhibiting predominantly mouth breathing demonstrated a higher prevalence of anterior dental caries.
